# Are we really all in this together? The social patterning of mortality during the first wave of the COVID-19 pandemic in Belgium

**DOI:** 10.1186/s12939-021-01594-0

**Published:** 2021-12-18

**Authors:** Sylvie Gadeyne, Lucia Rodriguez-Loureiro, Johan Surkyn, Wanda Van Hemelrijck, Wilma Nusselder, Patrick Lusyne, Katrien Vanthomme

**Affiliations:** 1 Sociology Department, Interface Demography, Vrije Universiteit Brussel, Pleinlaan 2, 1050 Brussels, Belgium; 2 Netherlands Interdisciplinary Demographic Institute-KNAW/University of Groningen, Lange Houtstraat 19, The Hague, CV NL-2511 The Netherlands; 3 Department of Public Health, Erasmus MC, Dr. Molewaterplein 40, Rotterdam, GD 3015 The Netherlands; 4 Statbel, Directorate General Statistics - Statistics Belgium, North Gate - Boulevard du Roi Albert II, 16 - 1000 Brussels, Belgium

**Keywords:** COVID-19, Mortality, Inequalities, Socioeconomic and socio-demographic patterning

## Abstract

**Background:**

Belgium was one of the countries that was struck hard by COVID-19. Initially, the belief was that we were ‘all in it together’. Emerging evidence showed however that deprived socioeconomic groups suffered disproportionally. Yet, few studies are available for Belgium. The main question addressed in this paper is whether excess mortality during the first COVID-19 wave followed a social gradient and whether the classic mortality gradient was reproduced.

**Methods:**

We used nationwide individually linked data from the Belgian National Register and the Census 2011. Age-standardized all-cause mortality rates were calculated during the first COVID-19 wave in weeks 11-20 in 2020 and compared with the rates during weeks 11-20 in 2015-2019 to calculate absolute and relative excess mortality by socioeconomic and -demographic characteristics. For both periods, relative inequalities in total mortality between socioeconomic and -demographic groups were calculated using Poisson regression. Analyses were stratified by age, gender and care home residence.

**Results:**

Excess mortality during the first COVID-19 wave was high in collective households, with care homes hit extremely hard by the pandemic. The social patterning of excess mortality was rather inconsistent and deviated from the usual gradient, mainly through higher mortality excesses among higher socioeconomic groups classes in specific age-sex groups. Overall, the first COVID-19 wave did not change the social patterning of mortality, however. Differences in relative inequalities between both periods were generally small and insignificant, except by household living arrangement.

**Conclusion:**

The social patterning during the first COVID-19 wave was exceptional as excess mortality did not follow the classic lines of higher mortality in lower classes and patterns were not always consistent. Relative mortality inequalities did not change substantially during the first COVID-19 wave compared to the reference period.

## Background

Many European countries faced a first outbreak of the Coronavirus 2 (SARS-CoV-2 or COVID-19) during spring 2020. The pandemic has had tremendous consequences at different societal levels. The most direct impact of COVID-19 relates to its consequences in terms of morbidity and mortality. A commonly adopted message – at least in the beginning stage of the pandemic – was that ‘COVID-19 did not discriminate’ [1]. That, being an infectious disease, it struck people without distinguishing classes and borders. This refers to an old belief; since infectious diseases dominated mortality patterns in the Ancient Regime, it was believed that socioeconomic (SE) inequalities in mortality did not occur during this era [2–4]. Related to COVID-19, it was also believed that mortality hit the higher SE classes more during the first wave. Skiing holidays and travelling for leisure and work-related goals – more typical for and affordable in the middle and higher classes – initially played an important role in the spread of COVID-19 [5], so one could expect a higher COVID-19 incidence and mortality in these SE groups.

Notwithstanding these initial thoughts, studies that linked COVID-19 related mortality to individual’s SE and socio-demographic (SD) characteristics showed a higher mortality in deprived SE groups [6–12], at advanced ages [8, 13–16], among males [8, 16–18] and among migrant minorities [8, 19, 20].

Based on these preliminary results and the huge evidence of SE inequalities in mortality [21–23], COVID-19 mortality can be expected to be higher in deprived SE classes. The underlying mechanisms are numerous [24] and include connections to the main dimensions of socioeconomic status (SES):

- Lower educational level may be associated with a lower accessibility to sound coping mechanisms paving the way to more risky behaviour such as smoking and poor dietary patterns defeating the immune system; to a higher frequency of co-morbidities (diabetes, obesity, respiratory diseases) increasing the risk of infection; with limited knowledge about how to implement COVID-19 lockdown and hygiene measures; and with poorer healthcare. Indirectly, education also influences the occupational position and income level that both relate to survival chances as well.
- Occupation may increase exposure to the virus, especially for individuals working in sectors characterised by constant human contact (bus drivers, healthcare workers, care home workers, retail staff, cleaners, teachers, etc.) compared to those working in sectors that allow working from home or in a more protected environment.
- Low income may affect living conditions in many ways, influencing the neighbourhood in which one is living, leading to poorer housing conditions and poorer dietary patterns. These aspects are in their turn associated with higher infection risks and with poorer access to efficient means to protect oneself and to cope with lockdown measures (protective material, a garden, living near green spaces and parks, …).

In addition, specific sociodemographic groups can be expected to have a higher risk of dying from COVID-19.

- Migrant groups more often belong to vulnerable SE classes and may be more difficult to reach with informative campaigns about protection measures and seeking health care due to the lack of efficient communication channels and to cultural or language barriers.
- Household living arrangement is another potential marker for COVID-19 related mortality: people living in single households may dispose of less social capital to act appropriately when infected but may otherwise be less exposed to the risk of infection by other household members. The difference between private and collective households, particularly residence in care homes at old age, is crucial as well, given the very high mortality in homes for the elderly [25].

The aim of this paper is to unravel the social patterning of mortality during the first COVID-19 wave in Belgium. First, we will look into excess mortality by SE/SD indicators in order to disentangle how this excess is patterned socioeconomically and socio-demographically. To our knowledge, only one study investigated COVID-19 excess mortality in Belgium, focussing on income inequalities [7], generally showing higher excess mortality with lower income groups. Second, we will compare relative mortality inequalities during the first COVID-19 wave with relative inequalities in a reference ‘normal’ period to dig deeper into the impact of COVID-19 on relative social inequalities in mortality. This issue has hardly been tackled in literature. Analyses integrate linked individual data on all-cause mortality and SE and SD indicators. Information is included on income, educational level, age, sex, migrant origin, household living arrangement, type of household and co-morbidity.

## Data and methods

### Design and study population

Data were provided by Statistics Belgium and consisted of a record linkage between different administrative data sources: the Belgian National Register (providing yearly population stocks with information on SD characteristics and vital status for all people officially residing in Belgium on the 1st of January of each year), the administrative census 2011 (providing education and housing data) and the tax register (providing yearly income data). The anonymised data were exhaustive and consisted of more than 11 million Belgians for whom a wide set of SE and SD variables were available. Analyses were performed for the entire population aged 25 and older.

### Variables

#### Mortality indicators

In order to investigate inequalities in COVID-19 related mortality, we approached mortality from different angles. First, we focussed on excess mortality by SE/SD characteristics. Mortality excess was defined as the higher (absolute and relative) age-standardised mortality rates during the first COVID-19 wave – i.e. from weeks 11-20 thus covering the period between the 9th of March and the 17th of May – compared to pre-COVID-19 mortality during a reference period, assumed to be characteristic for a normal mortality regime. The focus on excess mortality was justified by the fact that testing capacity and testing strategy have had a huge impact on the registration of deaths as COVID-19 deaths and the fact that data were not available yet. To assess the impact that COVID-19 has had on existing mortality inequalities, we secondly compared relative SE/SD inequalities in total mortality observed during the first COVID-19 wave with inequalities observed during the standard pre-COVID-19 reference period. Ideally, such a comparison should be limited to COVID-19 deaths, but cause-specific data are not available yet.

#### SD and SE variables

To gain clear insight into inequalities in COVID-19 related mortality, we focused on a range of SE and SD indicators. The use of different SE indicators is important as they represent different forms of SE (dis)advantage, and in addition they are formed during different phases of the life course [26, 27]. We included educational attainment and income as SE indicators. Education primarily represents the knowledge-related assets of a person and was classified in this study using the International Standard Classification of Education: primary education (0-1), lower secondary education (ISCED 2), higher secondary education (ISCED 3–4) and tertiary education (ISCED 5–6). Income captures current economic resources and was based on the total net taxable income per person, divided into deciles categorized as low income (deciles one to four), mid income (five to seven) and high income (eight to ten). As missing values may not be randomly distributed in the population, they were treated as separate categories, both for educational attainment and income level.

We included age, gender, household living arrangement, migrant background and home care residency as SD indicators. Household living arrangement partially reflects the social network and was operationalized by distinguishing persons living without a partner, persons living with a partner, other positions (children living with parents, multigenerational families…) and collective households. Collective households consist of elderly care homes, where people officially reside because of poor health and insufficient supportive networks, as well as all other residential facilities for disabled people or people with mental health problems and prisons, where people reside on a permanent basis. Migrant background was based on nationality of origin and distinguished Belgians, first-generation (FG) and second-generation (SG) migrants. We decided not to include a more detailed classification of the migrant community in Belgium as this was beyond the scope of this paper. In addition, a paper by Vanthomme et al. [20] investigated this topic of excess mortality by migrant background thoroughly in Belgium, using a more detailed variable.

As literature showed that co-morbidity played a significant role in COVID-19 related mortality, a proxy of co-morbidity prior to the COVID-19 pandemic was included. More specifically, individuals were categorised as having a chronic disease when their total taxable income in 2020 mainly consisted of sickness benefits (60% or more). As this indicator was based on income out of labour, it could only be constructed for the population younger than 65 years.

#### Statistical analysis

To obtain a measure of mortality excess during the first COVID-19 wave, we compared (directly) age-standardised mortality rates (ASMRs) calculated for weeks 11-20 in 2020 with the ASMRs calculated for the same weeks in 2015-2019, using the Belgian population of 2020 as the standard population. To obtain a robust and stable estimation of mortality in the reference period, geometric means of the ASMRs during weeks 11-20 of the various years (2015, 2016, 2017, 2018 and 2019) were calculated [28]. Absolute excess mortality was calculated as the absolute difference between both series of ASMRs. In addition, we calculated the relative (percentage) change between the ASMRs in 2020 and those in the standard period (relative to the standard period). To describe SD and SE inequalities, excess mortality (absolute and percentage change) was calculated by education, income, household living arrangement, migrant background and co-morbidity.

Second, to study whether the first COVID-19 wave altered the existing SE and SD inequalities in total mortality, we compared the age-adjusted mortality rate ratios (MRR) of both periods. MRRs were calculated for all SE and SD variables, using the overall population as reference, by running Poisson regression models with the log of the person-time as the offset. SE and SD variables were added one after the other. As we wanted to compare the gradients over the different subgroups, we decided not to use a stepwise selection procedure. In this way, we assured that each variable was added at the same time for each subgroup. In building the models, we followed a life course approach – first adding the socioeconomic variables achieved quite early in life through education and subsequently income; then the socio-demographic variables; and finally chronic disease – rather than a variable selection based on explanatory power or model fit. As many socioeconomic and socio-demographic variables are interrelated, we controlled for multicollinearity in a correlation matrix (results not shown). This matrix showed that socioeconomic and socio-demographic variables were correlated, but not in a way to jeopardize our results due to multicollinearity (the highest correlation was observed between education and income with a coefficient of 0,54, the second highest correlation was observed between living situation and income with a coefficient of 0.44).

To construct a robust picture of the standard period, we estimated MRRs for each year and calculated the geometric mean of the MRRs of the various years (2015-2019). The significance of the change over time was formally tested as explained by Altman and Bland [29]. All analyses were performed with Stata 14.2. Analyses were stratified by age (25-64, 65-84 and 85+) and sex, given the age-and sex-driven (COVID-19) mortality patterns and the fact that specific variables were available for analysis in the youngest groups only (chronic morbidity and migrant background). In the older age groups, we also stratified by care home residence as COVID-19 struck care homes very hard in Belgium.

## Results

### SE inequalities in excess mortality during the first wave of COVID-19 in Belgium

Table 1shows excess mortality expressed as excess deaths per 100,000 person-years and the percentage change as compared to the standard period (2015-2019) by age group, care home residency and SE/SD indicators. The results show that mortality was significantly higher during the first wave of the epidemic, with the largest excess mortality and percentage changes for the oldest age groups (65-84 and 85+). Excess mortality was also higher in men than in women in all age groups.

**Table 1:** Excess mortality per 100,000 person-years with 95% confidence intervals and % change in age-standardised mortality rate in the Belgian population during the first wave of COVID-19 in 2020 compared to the period 2015–2019 by gender, age group, care home and demographic and socioeconomic indicators

	25–64 years		65–84 years				85+ years			
			No care home		Care home		No care home		Care home	
	Absolute	%	Absolute	%	Absolute	%	Absolute	%	Absolute	%
**Men**										
**Total**	10.4 (6.7–14.2)	3.9	**254.3 (238.5–270.0)**	**24.1**	**5348.0 (4846.5–5849.6)**	**56.9**	**183.5 (164.7–202.2)**	**22.4**	**1550.5 (1482.8–1618.2)**	**49.7**
**Educational level**										
Primary or less	6.8 (−36.3–50.0)	1.6	251.2 (208.0–294.4)	22.8	**5128.8 (4185.6–6071.9)**	**55.2**	**198.2 (161.8–234.7)**	**22.8**	**1671.9 (1570.0–1773.7)**	**49.9**
Lower secondary	26.9 (4.9–48.7)	7.7	173.6 (136.9–210.2)	18.9	**5257.4 (4226.9–6288.0)**	**55.2**	**146.7 (100.6–192.9)**	**19.5**	**1318.3 (1152.7–1484.0)**	**45.5**
Upper secondary	1.2 (−10.2–12.7)	0.5	136.4 (96.2–176.7)	16.6	**5472.9 (4297.0–6648.8)**	**57.7**	**266.9 (208.5–325.4)**	**32.1**	**1900.3 (1728.0–2072.6)**	**57.3**
Higher education	4.7 (−4.4–13.9)	3.0	107.4 (73.3–141.5)	16.3	**4410.4 (2962.4–5858.3)**	**57.6**	**169.5 (111.7–227.4)**	**23.7**	**1092.6 (862.20–1323.0)**	**49.1**
Missing	18.9 (−12.0–50.0)	5.6	224.1 (145.6–302.4)	20.1	**6566.1 (5345.9–7786.4)**	**63.1**	**231.4 (151.1–311.6)**	**25.2**	**2023.5 (1869.3–2177.8)**	**56.0**
**Income level**										
Low	20.4 (− 2.3–43.1)	4.5	188.9 (156.4–221.4)	17.5	**5220.0 (4566.0–5873.9)**	**56.8**	**190.3 (159.0–221.4)**	**22.4**	**1693.8 (1601.5–1786.1)**	**50.8**
Middle	−15 (−28.9–−1.4)	−6.1	171.7 (146.9–196.7)	20.0	**6201.5 (5241.8–7161.2)**	**58.7**	**203.3 (168.8–237.7)**	**25.5**	**1585.3 (1469.3–1701.3)**	**51.3**
High	**21.0 (16.0–26.0)**	**14.1**	**110.1 (69.7–150.4)**	**17.5**	**6161.6 (4172.8–8150.6)**	**68.2**	**201.3 (137.7–264.9)**	**25.4**	**1105.3 (876.41–1334.3)**	**45.4**
Missing	2.7 (−61.7–67.2)	0.5	178.1 (−33.6–390.1)	16.4	**3736.7 (2105.9–5367.4)**	**64.2**	−160.4 (− 359.2–38.3)	−40.2	**1870.5 (1354.4–2386.6)**	**51.2**
**Living situation**										
With partner	3.2 (0.0–6.5)	1.9	129.3 (113.1–145.6)	16.4			**182.3 (156.4–208.2)**	**24.1**		
Without partner	−5.3 (−28.6–17.9)	−1.2	228.2 (182.4–274.2)	19.2			**218.1 (182.1–254.0)**	**24.5**		
Other	**62.7 (9.4–115.9)**	**14.5**	**246.1 (99.8–392.4)**	**20.2**			**183.4 (42.8–324.0)**	**17.6**		
Collective HH	**479.3 (113.0–845.7)**	**24.1**	**1469.4 (985.8–1952.9)**	**58.8**						
**Migrant background**										
Belgian	1.6 (−3.9–7.26)	0.6								
FG non-Belgian	**55.4 (35.8–74.9)**	**21.9**								
SG non-Belgian	25.2 (−14.7–65.2)	8.5								
**Chronic disease**										
No	**23.0 (19.9–26.0)**	**10.3**								
Yes	− 110.8 (−210.6–−11.1)	−11.5								
**Women**										
**Total**	4.2 (3.7–4.9)	2.7	**92.3 (85.0–99.7)**	**18.2**	**3027.4 (2623.5–3431.2)**	**52.5**	**129.7 (120.3–139.1)**	**22.2**	**843.8 (808.9–878.6)**	**42.0**
**Educational level**										
Primary or less	**70.0 (32.6–107.4)**	**23.4**	**126.8 (102.9–150.8)**	**21.3**	**2583.4 (1887.4–3279.4)**	**47.3**	**152.1 (132.5–171.6)**	**24.2**	**922.4 (866.9–977.8)**	**43.7**
Lower secondary	12.3 (−7.2–31.8)	5.8	**115.0 (93.6–136.3)**	**22.4**	**3299.9 (2435.4–4164.4)**	**52.9**	**138.1 (112.8–163.5)**	**24.7**	**914.7 (834.3–995.1)**	**45.1**
Upper secondary	−7.0 (−13.3–−0.7)	−5.1	**59.5 (34.3–84.9)**	**14.1**	**3036.0 (2116.9–3955.1)**	**54.5**	**116.5 (79.5–153.5)**	**22.5**	**743.1 (623.3–862.9)**	**40.3**
Higher education	0.9 (−4.2–6.1)	0.9	**57.8 (31.7–83.9)**	**15.5**	**2931.3 (1673.5–4189.1)**	**56.3**	**147.4 (97.3–197.3)**	**28.8**	**643.1 (498.2–787.9)**	**40.1**
Missing	28.4 (3.9–53.0)	13.1	**147.6 (96.3–198.8)**	**23.5**	**3775.2 (2716.1–4834.5)**	**62.0**	**122.0 (78.7–165.2)**	**20.7**	**818.0 (708.0–928.0)**	**41.0**
**Income level**										
Low	−2.2 (−8.2–3.6)	−1.2	**98.5 (86.9–110.1)**	**18.7**	**2999.8 (2495.5–3504.0)**	**52.4**	**122.7 (107.8–137.6)**	**20.7**	**835.3 (784.2–886.4)**	**41.3**
Middle	14.0 (4.6–23.6)	9.8	**100.8 (78.4–123.2)**	**19.9**	**3340.6 (2415.1–4266.1)**	**52.9**	**150.8 (128.9–172.7)**	**26.5**	**922.9 (862.0–983.9)**	**45.2**
High	2.0 (−2.7–7.0)	2.6	**63.7 (30.5–96.8)**	**17.3**	**4356.8 (2443.5–6270.1)**	**73.1**	**169.5 (118.5–220.5)**	**29.6**	**783.4 (645.7–921.2)**	**43.0**
Missing	37.8 (−13.1–88.7)	9.5	113.1 (−3.04–229.5)	18.9	**2513.8 (900.8–4126.8)**	**55.6**	**173.3 (21.4–325.3)**	**27.7**	**636.9 (222.7–1051.2)**	**38.2**
**Living situation**										
With partner	−4.7 (−4.9–−4.3)	−4.2	**72.1 (59.8–84.3)**	**16.5**			**98.5 (69.4–127.5)**	**20.0**		
Without partner	4.8 (−6.8–16.5)	2.2	**107.9 (90.8–125.0)**	**19.1**			**131.7 (119.8–143.6)**	**23.5**		
Other	30.8 (−17.–79.6)	10.9	**233.3 (149.6–317.0)**	**29.3**			**323.7 (254.9–392.5)**	**32.1**		
Collective HH	**1251.8 (628.9–1874.8)**	**48.9**	**718.6 (330.8–1106.4)**	**54.0**						
**Migrant background**										
Belgian	1.3 (−0.8–3.5)	0.8								
FG non-Belgian	17.5 (6.3–28.6)	12.7								
SG non-Belgian	12.7 (−15.9–41.5)	7.2								
**Chronic disease**										
No	7.1 (6.9–7.4)	5.7								
Yes	−35.6 (−78.2–6.9)	−7.5								

Results in Bold show where the ASMR’s are significantly different (*p* < 0.05) in 2020 compared with the standard period

*HH* Household, *FG* First generation, *SG* Second generation

Looking at the results by age group, there was a significant level of excess mortality during the first COVID-19 wave among low-educated women (23%), high-income men (14%), non-Belgian first-generation men (22%) and among men without chronic morbidity (10%) in the youngest age group (aged 25-64). Men and women living in a collective household were affected extremely hard in terms of mortality during the first wave of COVID-19: for men a 24% excess mortality was observed, and for women even 49%. In absolute terms, 1993 per 100,000 men living in a collective household died during the COVID-19 observation period in contrast to 1513 men per 100,000 during the same weeks in the standard period. For women, these numbers increased from 1306 per 100,000 in 2019 to 2558 per 100,000 in 2020.

For the group aged 65-84 years, we generally found significant and large mortality excesses for all SE/SD subgroups. A striking result was the difference between individuals who did and those who did not live in care homes: excess mortality was ‘limited’ to 24% (or 254 deaths per 100,000 person-years) for male and 18% (or 92 surplus deaths) for female non-residents compared to 57% (or 5348 deaths per 100,000 person-years) for male and 53% (or 3027 deaths) for female care home residents. Absolute and relative mortality excesses in this age group did not vary consistently by education and income among men, although middle- and high-income groups seemed characterised by a higher excess mortality with resident men. Among women not residing in care homes, we found the largest mortality excesses among those with unknown and lower (primary or less and lower secondary) educational attainment, whereas the opposite was observed among care home residents. We detected a similar pattern by income (although not extending to the missing category). By household living arrangement, non-resident men and women cohabitating with someone other than their partner showed a mortality surplus of respectively 20 and 29%.

For the oldest age group (85 years and older), mortality during the first COVID-19 wave was again consistently higher among all SE/SD subgroups compared with the reference period. Once more, there was a large difference by care home residency with residents showing a much higher excess mortality during the first COVID-19 wave compared to elderly people who still lived independently: a 50% versus 22% higher mortality level among men (or 1551 versus 184 additional deaths per 100,000), and a 42% versus 22% difference among women (or 844 versus 130 deaths per 100,000). By education, excess mortality was highest for men with an upper secondary degree and the missing category, while for income, patterns varied when considering absolute and relative excess mortality. For women, the largest excess was observed for the lower and lower secondary educated and middle-income among care home residents. In non-resident women, patterns by education were inconsistent, while by income excesses were largest in the high-income and missing categories.

### Change in relative SE inequalities in mortality during the first wave of COVID-19

Table 2 shows that relative SD and SE inequalities in total mortality generally followed a classic mortality gradient during the first wave of COVID-19, whereby levels were higher in more deprived classes, both for men and women. However, for several indicators the associations were slightly weaker compared with the standard period.

**Table 2:** Relative inequalities in mortality during the first COVID-19 wave among the Belgian population aged 25–64 years, by gender

	Model 1		Model 2		Model 3		Model 4		Model 5	
	MRR	95% C.I.	MRR	95% C.I.	MRR	95% C.I.	MRR	95% C.I.	MRR	95% C.I.
**Men - 2020**										
**Age at census**	1.10	1.09–1.10	1.10	1.09–1.10	1.10	1.09–1.10	1.10	1.09–1.10	1.09	1.09–1.10
**Educational level**										
Primary or less	1.51	1.37–1.67	1.28	1.15–1.41	1.29	1.17–1.43	1.23	1.11–1.36	1.18	1.06–1.30
Lower secondary	1.21	1.12–1.31	1.23	1.13–1.33	1.12	1.03–1.22	1.14	1.05–1.24	1.13	1.04–1.22
Upper secondary	0.87	0.81–0.94	1.00	0.93–1.09	0.91	0.84–0.99	0.94	0.87–1.03	0.96	0.88–1.04
Higher education	0.56	0.51–0.62	0.76	0.69–0.85	0.72	0.64–0.80	0.75	0.67–0.84	0.78	0.70–0.87
Missing	1.13	1.02–1.25	0.83	0.75–0.93	1.06	0.93–1.20	1.01	0.89–1.14	1.01	0.89–1.14
**Income level**										
Low			1.38	1.28–1.47	1.41	1.32–1.51	1.38	1.29–1.48	1.12	1.03–1.21
Middle			0.74	0.68–0.80	0.72	0.67–0.79	0.81	0.75–0.88	0.80	0.74–0.87
High			0.50	0.46–0.55	0.48	0.44–0.52	0.57	0.52–0.62	0.62	0.57–0.68
Missing			1.95	1.75–2.17	2.05	1.84–2.29	1.57	1.40–1.75	1.80	1.61–2.01
**Migrant background**										
Belgian					1.28	1.18–1.39	1.21	1.11–1.31	1.20	1.11–1.30
FG non-Belgian					0.69	0.62–0.77	0.78	0.70–0.86	0.80	0.72–0.89
SG non-Belgian					1.14	1.02–1.28	1.07	0.95–1.20	1.04	0.93–1.17
**Living situation**										
With partner							0.45	0.41–0.48	0.48	0.44–0.52
Without partner							0.97	0.89–1.05	0.99	0.91–1.07
Other							0.83	0.74–0.93	0.89	0.79–1.00
Collective HH							2.79	2.38–3.27	2.37	2.03–2.77
**Chronic disease**										
No									0.63	0.59–0.67
Yes									1.58	1.49–1.68
Likelihood-ratio test	1979.89		399.63		83.29		380.62		231.85	
Prob > chi^2^	0.0000		0.0000		0.0000		0.0000		0.0000	
**Women - 2020**										
**Age at census**	1.10	1.09–1.11	1.10	1.09–1.11	1.10	1.09–1.11	1.10	1.09–1.10	1.09	1.09–1.10
**Educational level**										
Primary or less	1.59	1.41–1.81	1.45	1.27–1.64	1.48	1.30–1.68	1.36	1.19–1.54	1.30	1.14–1.47
Lower secondary	1.11	1.00–1.24	1.10	0.98–1.22	0.96	0.86–1.08	0.99	0.88–1.11	0.97	0.86–1.08
Upper secondary	0.79	0.71–0.87	0.86	0.77–0.95	0.74	0.67–0.83	0.78	0.70–0.88	0.80	0.71–0.89
Higher education	0.60	0.54–0.68	0.82	0.72–0.93	0.74	0.64–0.84	0.80	0.70–0.91	0.84	0.73–0.96
Missing	1.19	1.04–1.37	0.90	0.78–1.05	1.29	1.07–1.55	1.19	1.00–1.42	1.20	1.01–1.42
**Income level**										
Low			1.02	0.93–1.12	1.03	0.94–1.13	1.19	1.08–1.30	0.92	0.83–1.02
Middle			0.85	0.76–0.95	0.82	0.73–0.92	0.90	0.80–1.01	0.86	0.77–0.97
High			0.52	0.45–0.60	0.49	0.42–0.56	0.54	0.46–0.62	0.60	0.51–0.69
Missing			2.22	1.92–2.55	2.42	2.09–2.79	1.74	1.49–2.03	2.12	1.81–2.47
**Migrant background**										
Belgian					1.34	1.20–1.50	1.29	1.16–1.43	1.29	1.16–1.43
FG non-Belgian					0.58	0.49–0.68	0.65	0.56–0.76	0.67	0.58–0.78
SG non-Belgian					1.29	1.11–1.50	1.19	1.03–1.39	1.16	1.00–1.35
**Living situation**										
With partner							0.37	0.33–0.41	0.42	0.37–0.46
Without partner							0.74	0.67–0.83	0.74	0.67–0.83
Other							0.80	0.68–0.94	0.84	0.71–0.99
Collective HH							4.52	3.67–5.57	3.83	3.14–4.68
**Chronic disease**										
No									0.57	0.53–0.61
Yes									1.76	1.64–1.90
Likelihood-ratio test	1265.05		135.76		82.92		299.17		230.52	
Prob > chi^2^	0.0000		0.0000		0.0000		0.0000		0.0000	

*MRR* Mortality rate ratio, *C.I.* Confidence interval

**Fig. 1:**
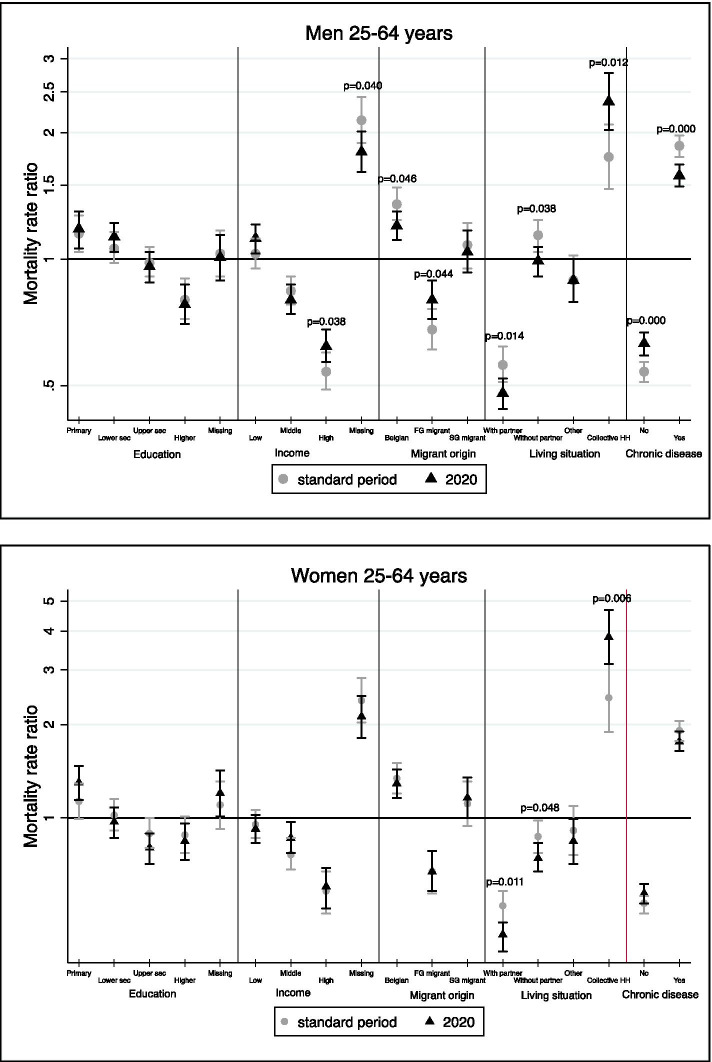
Change in relative inequalities in mortality between the first COVID-19 wave and the standard period (2015-2019) among the Belgian population aged 25-64 years, by gender. Results of the full-adjusted models

In the youngest age group (25-64 years), for example, we found a somewhat smaller income gradient during the first COVID-19 wave compared to the reference period among men (Fig. 1). To illustrate, in the standard period, the high-income group had 46% lower mortality compared with the overall mean (MRR: 0.54; 95% C.I.: 0.49-0.60), whereas in 2020 their MRR was 38% lower (95% C.I.: 0.57-0.68). Similarly, for migrant background, the mortality advantage of first-generation migrant men and also the mortality disadvantage for Belgian men declined (when controlling for education and income). Among men, mortality differences were less pronounced for the variable ‘co-morbidity’ as well: men with co-morbidity showed a lower excess mortality than before (a MRR of 1.58 (95% C.I.: 1.49-1.68) in 2020 compared to 1.86 in the standard period (95% C.I.: 1.75-1.97), while those without co-morbidity showed a smaller advantage in the full-adjusted model. In contrast to these weaker relative mortality differences during the first COVID-19 wave, we observed a stronger pattern by household living arrangement for both men and women.

**Fig. 2:**
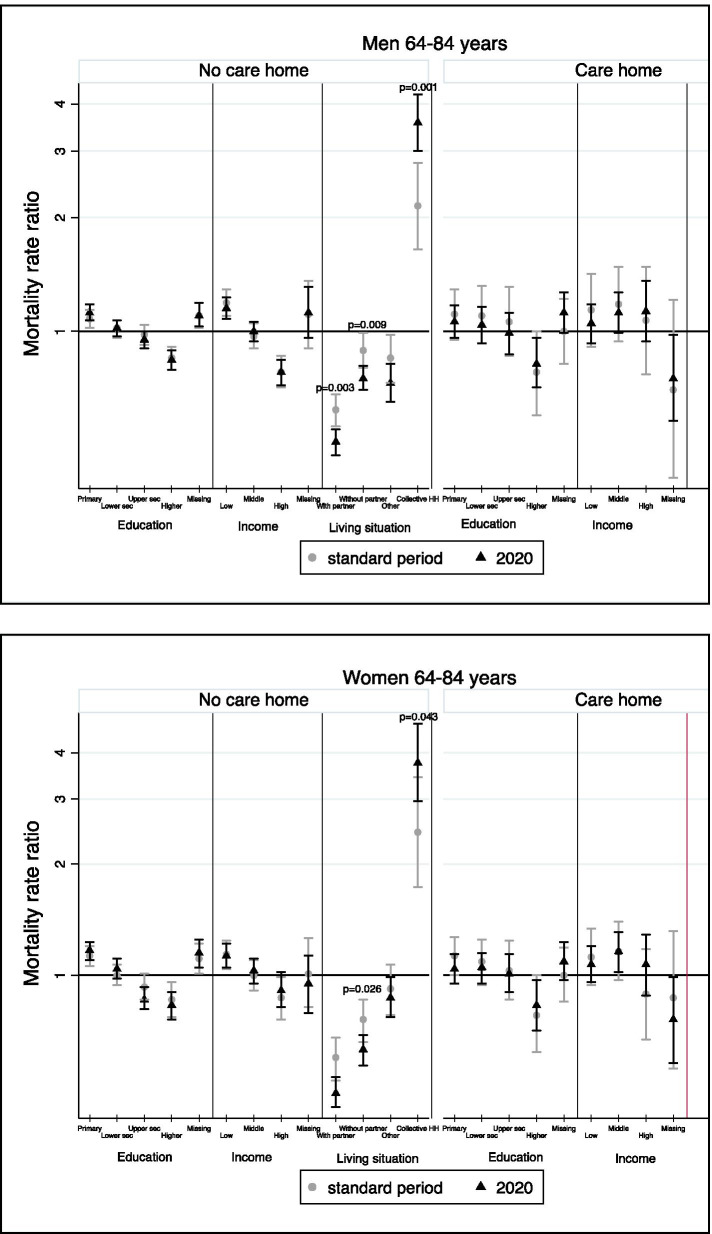
Change in relative inequalities in mortality between the first COVID-19 wave and the standard period (2015-2019) among the Belgian population aged 65-84 years, by gender and care home. Results of the full-adjusted models

In the group aged 65-84 years, we observed significant SE and SD inequalities among men and women who still live independently, but mostly insignificant coefficients for men and women living in a care home (Table 3). Regarding the changes in the relative inequalities during the first COVID-19 wave compared to the standard period, we found significant changes for household living arrangement only (Fig. 2). This inequality was indeed significantly larger during the pandemic in 2020 than during the standard period (Fig. 2). The most striking result was that for respondents living in a collective household: in the standard period, men and women showed a MRR of 2.15 (95% C.I.: 1.65-2.79) and 2.44 (95% C.I.: 1.73-3.44) respectively, which increased during the first COVID-19 wave of 2020 to 3.57 (95% C.I.: 3.00-4.24) and 3.76 (95% C.I.: 2.96-4.79) respectively.

**Table 3:** Relative inequalities in mortality during the first COVID-19 wave among the Belgian population aged 65–84 years, by gender and care home

	No care home						Care home			
	Model 1		Model 2		Model 3		Model 1		Model 2	
	MRR	95% C.I.	MRR	95% C.I.	MRR	95% C.I.	MRR	95% C.I.	MRR	95% C.I.
**Men**										
**Age at census**	1.10	1.10–1.11	1.10	1.09–1.10	1.10	1.10–1.11	1.05	1.04–1.06	1.05	1.04–1.06
**Educational level**										
Primary or less	1.19	1.14–1.25	1.13	1.08–1.19	1.12	1.07–1.18	1.05	0.96–1.15	1.06	0.96–1.17
Lower secondary	1.02	0.97–1.07	1.00	0.96–1.05	1.02	0.97–1.07	1.05	0.94–1.16	1.04	0.93–1.16
Upper secondary	0.91	0.86–0.96	0.93	0.88–0.98	0.95	0.90–1.00	1.00	0.88–1.13	0.99	0.87–1.12
Higher education	0.73	0.69–0.77	0.82	0.77–0.87	0.84	0.79–0.89	0.84	0.73–0.97	0.82	0.71–0.96
Missing	1.23	1.15–1.32	1.16	1.08–1.25	1.10	1.03–1.19	1.08	0.96–1.22	1.12	0.99–1.27
**Income level**										
Low			1.12	1.05–1.19	1.15	1.08–1.23			1.05	0.93–1.18
Middle			0.95	0.89–1.01	1.00	0.94–1.06			1.12	0.99–1.27
High			0.74	0.68–0.80	0.78	0.72–0.84			1.13	0.94–1.36
Missing			1.28	1.10–1.49	1.12	0.96–1.31			0.75	0.58–0.98
**Living situation**										
With partner					0.51	0.47–0.55				
Without partner					0.75	0.70–0.81				
Other					0.73	0.65–0.82				
Collective HH					3.57	3.00–4.24				
Likelihood-ratio test	2142.58		89.10		331.71		78.51		5.32	
Prob > chi^2^	0.0000		0.0000		0.0000		0.0000		0.1499	
**Women**										
**Age at census**	1.11	1.10–1.11	1.11	1.10–1.11	1.10	1.10–1.11	1.03	1.01–1.04	1.02	1.01–1.04
**Educational level**										
Primary or less	1.20	1.14–1.26	1.18	1.12–1.25	1.17	1.10–1.23	1.04	0.96–1.13	1.04	0.95–1.14
Lower secondary	1.04	0.98–1.10	1.03	0.97–1.09	1.04	0.98–1.11	1.06	0.96–1.16	1.05	0.95–1.15
Upper secondary	0.85	0.79–0.91	0.85	0.79–0.91	0.86	0.81–0.93	1.02	0.91–1.15	1.01	0.90–1.14
Higher education	0.76	0.70–0.82	0.79	0.73–0.86	0.83	0.76–0.90	0.84	0.72–0.96	0.83	0.71–0.97
Missing	1.25	1.15–1.36	1.22	1.12–1.33	1.15	1.05–1.25	1.06	0.95–1.20	1.09	0.97–1.23
**Income level**										
Low			1.01	0.94–1.09	1.13	1.05–1.22			1.07	0.96–1.20
Middle			1.02	0.94–1.10	1.03	0.95–1.11			1.16	1.02–1.31
High			0.88	0.79–0.98	0.91	0.82–1.02			1.07	0.88–1.29
Missing			1.11	0.94–1.31	0.95	0.79–1.13			0.76	0.58–0.99
**Living situation**										
With partner					0.48	0.44–0.53				
Without partner					0.63	0.57–0.69				
Other					0.87	0.77–0.99				
Collective HH					3.76	2.96–4.79				
Likelihood-ratio test	1625.45		5.41		179.05		27.50		6.45	
Prob > chi^2^	0.0000		0.1439		0.0000		0.0000		0.0916	

*MRR* Mortality rate ratio, *C.I.* Confidence interval

**Fig. 3:**
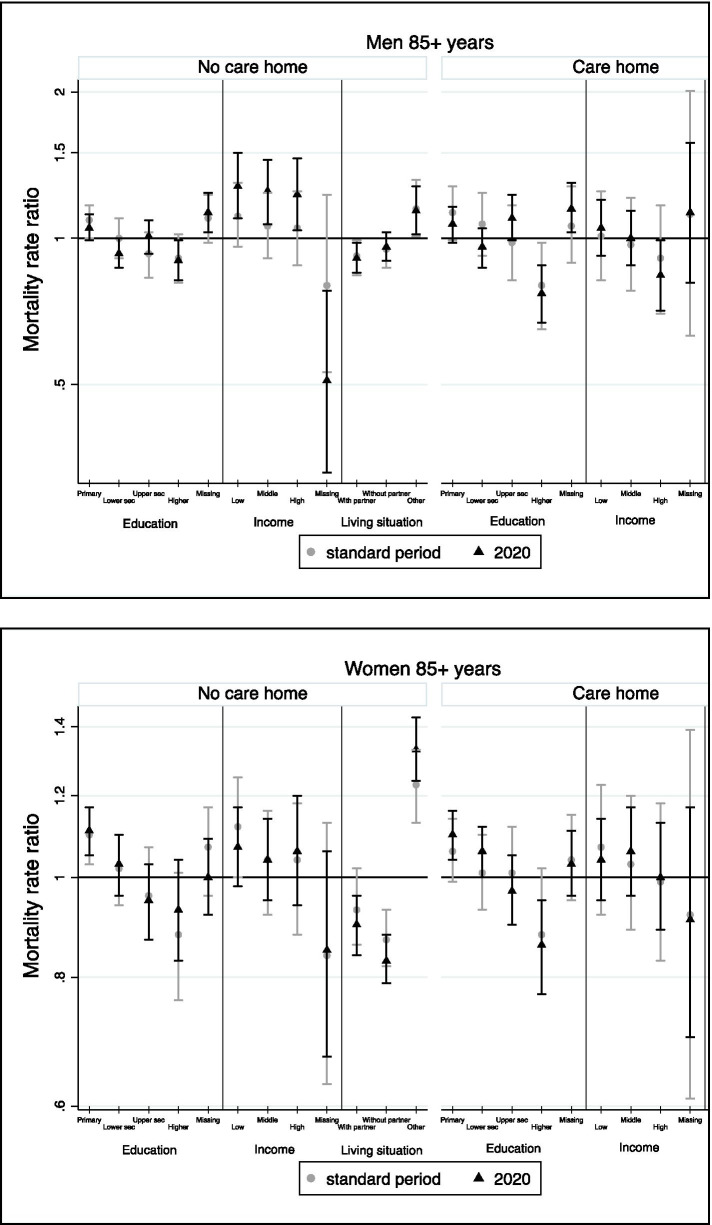
Change in relative inequalities in mortality between the first COVID-19 wave and the standard period (2015-2019) among the Belgian population aged 85+ years, by gender and care home. Results of the full-adjusted models

Within the group of 85-plussers, none of the associations were significantly stronger or smaller during the observation period in 2020 compared with the standard period (Fig. 3). In men, most categories of the SD and SE indicators were significantly associated with mortality for those living independently, while for those in care homes only the higher educated and income groups showed significantly lower MRRs compared to the mean (Table 4). In women, income did not generate any significant differences. For education on the other hand, we found that lower educated women showed a mortality excess both among care home residents and non-residents.

**Table 4:** Relative inequalities in mortality during the first COVID-19 wave among the Belgian population aged 85+ years, by gender and care home

	No care home						Care home			
	Model 1		Model 2		Model 3		Model 1		Model 2	
	MRR	95% C.I.	MRR	95% C.I.	MRR	95% C.I.	MRR	95% C.I.	MRR	95% C.I.
**Men**										
**Age at census**	1.10	1.09–1.12	1.10	1.09–1.12	1.10	1.09–1.12	1.04	1.02–1.05	1.04	1.02–1.05
**Educational level**										
Primary or less	1.07	1.01–1.14	1.06	0.99–1.13	1.05	0.99–1.12	1.10	1.02–1.19	1.07	0.98–1.16
Lower secondary	0.94	0.87–1.01	0.93	0.86–1.00	0.93	0.87–1.00	0.96	0.87–1.06	0.96	0.87–1.05
Upper secondary	1.01	0.93–1.09	1.00	0.93–1.09	1.01	0.93–1.09	1.08	0.97–1.20	1.10	0.99–1.23
Higher education	0.88	0.81–0.96	0.89	0.81–0.98	0.90	0.82–0.99	0.73	0.64–0.84	0.77	0.67–0.88
Missing	1.12	1.02–1.23	1.14	1.03–1.25	1.13	1.03–1.24	1.19	1.07–1.34	1.15	1.03–1.30
**Income level**										
Low			1.26	1.08–1.46	1.28	1.10–1.50			1.05	0.92–1.20
Middle			1.22	1.05–1.42	1.25	1.07–1.45			1.00	0.88–1.14
High			1.21	1.03–1.43	1.23	1.04–1.46			0.84	0.71–0.99
Missing			0.54	0.35–0.82	0.51	0.33–0.78			1.13	0.81–1.57
**Living situation**										
With partner					0.91	0.85–0.98				
Without partner					0.96	0.90–1.03				
Other					1.14	1.02–1.28				
Likelihood–ratio test	276.46		11.46		6.44		49.23		5.83	
Prob > chi^2^	0.0000		0.0095		0.0399		0.0000		0.1203	
**Women**										
**Age at census**	1.14	1.13–1.14	1.14	1.13–1.14	1.13	1.12–1.14	1.05	1.04–1.06	1.05	1.04–1.06
**Educational level**										
Primary or less	1.12	1.06–1.18	1.11	1.05–1.18	1.11	1.05–1.17	1.11	1.05–1.16	1.10	1.04–1.16
Lower secondary	1.02	0.96–1.09	1.02	0.96–1.09	1.03	0.96–1.10	1.06	1.00–1.13	1.06	1.00–1.12
Upper secondary	0.94	0.86–1.02	0.94	0.87–1.02	0.95	0.87–1.03	0.97	0.90–1.06	0.97	0.90–1.05
Higher education	0.92	0.83–1.02	0.93	0.83–1.04	0.93	0.83–1.04	0.85	0.77–0.94	0.86	0.77–0.95
Missing	1.02	0.94–1.11	1.01	0.93–1.10	1.00	0.92–1.09	1.03	0.96–1.11	1.03	0.96–1.11
**Income level**										
Low			1.03	0.94–1.12	1.07	0.98–1.17			1.04	0.95–1.14
Middle			0.98	0.89–1.07	1.04	0.95–1.14			1.06	0.96–1.17
High			0.99	0.88–1.11	1.06	0.94–1.20			1.00	0.89–1.13
Missing			1.01	0.81–1.26	0.85	0.67–1.06			0.91	0.70–1.17
**Living situation**										
With partner					0.90	0.84–0.96				
Without partner					0.83	0.79–0.88				
Other					1.33	1.24–1.43				
Likelihood-ratio test	732.75		1.78		78.17		163.66		1.49	
Prob > chi^2^	0.0000		0.6189		0.0000		0.0000		0.6855	

*MRR* Mortality rate ratio, *C.I.* Confidence interval

Most models showed significant goodness of fit statistics. The only exception was observed for the model adding income among men in care homes aged 65-84 and 85+ and among women aged 65-84 and 85 + .

## Discussion and conclusion

This paper aimed to investigate mortality excesses by SE/SD characteristics during the first COVID-19 wave in Belgium and the consequent changes in SE and SD mortality inequalities. Our findings showed that household living arrangement was the strongest discriminator of excess mortality during the first COVID-19 wave. High excess mortality was observed for residents of collective households, independent of age. However, at young ages (25-64), excess mortality did not follow a clear pattern by SE and SD characteristics. Mortality excesses were often not statistically significant at these ages. Exceptions were the excesses observed for high-income and non-Belgian first-generation migrant men and for low-educated women. In the older age groups, where excess mortality was much higher, SE differences in excess mortality were somewhat more pronounced. Overall, our results suggest that we were not all in this together, that the virus affected socioeconomic and demographic groups in different and sometimes unexpected ways.

In general, differences did not always follow clearly the consistent social gradient in health outcomes that is usually observed in Belgium and elsewhere, with deprived classes showing worse health outcomes notwithstanding the compulsory health insurance system. For income for instance, we observed higher excesses in the highest income group among men aged 25-64, in the middle and high-income groups for men and women aged 65-84 and among non-resident men and resident women aged 85+. The higher excess mortality among men 25-64 with a higher income might be explained by the higher infection risks through travelling for leisure and work-related goals in the active age group – more typical for and affordable in the middle and higher classes. In the older non-active population groups, intergenerational transmission from high-class children to high-class parents could have played a role in explaining these patterns.

It is important to underline that our study relates to the first COVID-19 wave. The observed higher excess mortality in higher income classes and/or the relatively small differences or inconsistent patterns of excess mortality for other covariates should be related to the early stage of the disease. The fundamental cause theory [30–32] and the stages of diseases theory [33] posit that inequalities in diseases evolve through different phases depending on the knowledge of and insights into risk factors, prevention and treatment of diseases. Given the fact that COVID-19 was a new infectious disease, these insights initially lacked, hence the inconsistent patterns. In this light, we can expect that inequalities will rise and progress to the well-established social gradient in mortality given the mechanisms of social health determination cited in the introduction. Future research should thus focus on the comparison of SE and SD excess mortality during the first wave and the later waves of COVID-19. Clouston et al. for instance showed that in the US, COVID-19 incidence and mortality was initially positively associated with SE characteristics, but subsequently the association inverted as public health measures were put in place [5].

Notwithstanding the atypical patterns in excess mortality, it seems that COVID-19 did not fundamentally alter the classic gradient of higher overall mortality rates with lower classes. Focussing on trends in relative mortality differences before and during the first COVID-19 wave, inequalities seem quite stable with lower social classes showing a higher mortality compared to higher classes. For some SE dimensions, for instance income, inequalities may seem somewhat smaller in 2020 for young men. This is not surprising given the observed higher excess mortality that was observed with specific higher-class categories. Similarly, by migration background, inequalities seem to have declined significantly among young men. Belgians showed a smaller mortality disadvantage and non-Belgian first-generation migrants a smaller advantage during the first COVID-19 wave.

Overall, however, differences in relative inequalities between 2020 and the reference period were not statistically significant. In addition, inequalities seemed larger in 2020 for some dimensions of SE position, for example for educational attainment among non-resident men aged 85+ (although not significantly) and for living situation. The patterns observed by household living arrangement in 2020 confirmed the common mortality gradient; people living with a partner showed the lowest rates and those without a partner, and especially those in collective households, the highest rates. The higher relative mortality of the latter in 2020 is one of the most striking results of this study.

Our analysis allowed for some interesting supplementary conclusions. Comparing care home residents and non-residents aged 65-84, mortality seemed less socially differentiated among residents especially with men. This could be related to the fact that residence in care homes is strongly related to the prevalence of chronic conditions at these ages, resulting in a resident population that is selected because of health problems [34–36]. Elderly people are living longer independently (with or without a partner or other persons in their private household) and are encouraged and supported by the government to do so. As a result, almost exclusively elderly people with serious health issues have been entering care homes. In such a population, we did not expect the social mortality differences that we observed in our study. In the population aged 85+, SE indicators generate more significant mortality differences compared to residents aged 65-84. It could be hypothesised that residency in care homes at these advanced ages is rather related to functional limitations and not so much to life threatening chronic illness conditions. However, we could not find any source that subscribed this hypothesis. In addition, we did not dispose of information on the age at which residents entered the care home and on their medical conditions at entrance.

Whatsoever, the significant social differences in mortality at these advanced ages (85+) were striking. In a selected population such as care home residents, we would expect that care home living conditions would eliminate SE differences. In this sense, our results underlined the persistence of social inequality in life chances with increasing age. The mechanisms behind these differences in care homes are particularly interesting for further investigation. In a life course perspective, the observed inequalities could result from accumulated factors over the course of elderly’s lives. Another promising venue would be to investigate if persisting social mortality differences in care homes could be related to the quality of the care offered in private versus public care homes. Residential care in Belgium is in hands of private companies or municipal care companies. A recent trend is the marketing and scaling up of care for the elderly, in which more and more municipalities wish to privatise their care companies.

Apart from social inequalities, excess mortality in care homes has been tremendously high. Mortality increased with 254 deaths (per 100.000 person-years) among non-resident men compared to 5348 deaths among resident men and with respectively 92 and 3027 deaths among non-resident and resident women. The sex difference in mortality in care homes, can be related to differential health selection between men and women into care homes: as care home residence depends both on health and on availability of networks, more unhealthy men than women enter into care homes. Whatsoever, the very large increase of mortality in care homes undoubtedly resulted from the lack of means and staff and the late governmental response to the COVID-19 pandemic whereby the pandemic could not be managed efficiently and timely in care homes. Lagasse et al. showed that the virus was present in Belgian care homes at the very beginning of the outbreak and that it was able to spread very rapidly within these facilities due to delayed governmental measures [37]. Amnesty International even concluded that fundamental human rights – such as the right to medical care – have been violated in care homes during the first COVID-19 wave [38]. It is clear that our figures call for more staff, preventive testing and social support in care homes.

In the active age groups aged 25-64, it was possible to control for a proxy for co-morbidity illness. Analyses revealed that persons with a pre-existing medical condition experienced higher mortality risks both among men and women, but that relative mortality inequalities were significantly smaller during the first pandemic wave. COVID-19 might have eradicated part of the differences in life chances between people with and people without co-morbidity.

This co-morbidity variable could only be calculated for the active age groups. Our study has several other limitations. At the time of the analyses, it was impossible to isolate deaths due to COVID-19. Consequently, we could not examine underlying mechanisms of the excess mortality during the first COVID-19 wave. Excess mortality however results from different counteracting causes of death: the decline of mortality for specific causes – such as traffic accidents – and the increase of other causes of death. There is a clear risk that mortality among non-infected persons has been affected, not in the least due to the fact that planned surgery and other medical consultations have been postponed and due to the reduced capacity to treat other acute conditions [39]. Hence, excess mortality should be interpreted as mortality related to the COVID-19 epidemic rather than people dying from COVID-19. Nevertheless, even if cause-specific data would become available, such data would need to be handled with caution due to the difficulties in registration of a decease as COVID-19 conditional upon the testing capacity during the first wave and the interplay between COVID-19 and other causes of death. Lagasse et al. [37] argued in this respect that excess mortality is a good indicator to estimate the total impact of COVID-19. For Belgium specifically, it has been shown that excess mortality and COVID-19 attributed mortality coincide perfectly, which strengthens our research results [37]. Furthermore, no data were available on occupation, health seeking behaviour and other determinants of mortality differences. A final limitation relates to the operationalisation of migrant background. Given the large number of covariates in our analysis, we decided to include a relatively rough variable that distinguishes Belgians from non-Belgian first- and second-generation migrants. In addition, due to the low number of migrants at older ages, especially in care homes, we could not include this variable in the older age groups. A paper by Vanthomme et al. [20] used a more detailed migrant background variable. Analyses showed that in middle-age mortality increased in all origin groups during the first COVID-19 wave, with significant excess mortality for Belgians and sub-Saharan African men. At old age, excess mortality was observed for all groups as well. In relative terms, most male elderly migrant groups showed higher mortality than natives, as opposed to 2019 and to women. Adding the sociodemographic and socioeconomic control variables decreased this excess mortality.

The strength of our study mainly relates to the exhaustive dataset that allowed for a detailed analysis of subgroups. All deaths in the country were captured in our dataset and we could include a wide variety of SE and SD variables some of which have played an important role in the spread of the COVID-19 virus. Our study is one of the first studies to dig into SE inequalities in Covid-19 excess mortality in Belgium. The earlier analysis of Decoster et al. [7] essentially focussed on income differences in the population aged 40-64 and 65+. They showed that the negative income gradient in mortality during the pandemic was comparable to the gradient in non-pandemic time in the younger population. For the elderly, slightly higher relative income differences were observed during the pandemic. Our paper partly corroborates these findings: the relative gradient did not change much, but we observed slightly lower relative differences by income in the population aged 25-64. This could be due to several reasons, mainly related to methodological issues, such as differing study populations and income variables, different methods of analysis and statistical models.

Future information on deaths during the second COVID-19 wave will reveal how different causes of death have interacted with COVID-19 mortality and through which other causes of death mortality excesses were generated. Notwithstanding the inconsistent patterning of excess mortality during the first COVID-19 wave, inequalities in overall mortality persisted and hardly changed. Policy makers should therefore keep in mind that a ‘one size fits all’-policy is not the best public health response for the future. Specific policy measures and communication strategies should be set-up that take the particular risks of population subgroups into account. Important in this regard would be e.g. assuring the safety at the workplace since deprived groups may be more likely to be employed in sectors that are not allowed working from home or in protected environments. Future research should without any doubt focus on the impact of the subsequent COVID-19 waves on mortality by SE/SD characteristics and compare results with what happened during the first wave.

## Data Availability

The analyses are based on data from a census-linked mortality follow-up study and cannot be made available due to privacy issues. Researchers can gain full access to the data by submitting an application to the Privacy Commission Belgium. In order to get permission to use data from the Belgian population register linked to census data an authorization request (in Dutch or French) needs to be submitted to the Belgian Data Protection Authority. The authorization request includes an application form and additional forms regarding data security. The necessary forms for the authorization request can be downloaded from the Data Protection Authority website (https://www.dataprotectionauthority.be). Next to information on the applicant and a list of requested data, the authorization request should specify why the data from the population register are necessary, for which time span data will be stored, and who will have access to the data. The codes for the analyses are available upon request (please contact the corresponding author).
